# Radiological imaging protection: a study on imaging dose used while planning computed tomography for external radiotherapy in Japan

**DOI:** 10.1093/jrr/rrad098

**Published:** 2023-12-26

**Authors:** Satoshi Kito, Yuhi Suda, Satoshi Tanabe, Takeshi Takizawa, Tomomasa Nagahata, Naoki Tohyama, Hiroyuki Okamoto, Takumi Kodama, Yukio Fujita, Hisayuki Miyashita, Kazuya Shinoda, Masahiko Kurooka, Hidetoshi Shimizu, Takeshi Ohno, Masataka Sakamoto

**Affiliations:** Division of Radiation Oncology, Department of Radiology, Tokyo Metropolitan Cancer and Infectious Diseases Center Komagome Hospital, 3-18-22 Honkomagome, Bunkyo-ku, Tokyo 113-8677, Japan; Department of Radiology, Tokyo Metropolitan Bokutoh Hospital, 4-23-15 Kotobashi, Sumida-ku, Tokyo 130-8575, Japan; Division of Radiation Oncology, Department of Radiology, Tokyo Metropolitan Cancer and Infectious Diseases Center Komagome Hospital, 3-18-22 Honkomagome, Bunkyo-ku, Tokyo 113-8677, Japan; Department of Radiology, Tokyo Metropolitan Bokutoh Hospital, 4-23-15 Kotobashi, Sumida-ku, Tokyo 130-8575, Japan; Department of Radiation Oncology, Niigata University Medical and Dental Hospital, 1-757 Asahimachi-dori, Chuo-ku, Niigata 951-8510, Japan; Department of Radiation Oncology, Niigata Neurosurgical Hospital, 3057 Yamada, Nishi-ku, Niigata 950-1101, Japan; Radiological Division, Osaka Metropolitan University Hospital, 1-5-7 Asahi-chou, Osaka City, Osaka 545-8586, Japan; Division of Medical Physics, Tokyo Bay Makuhari Clinic for Advanced Imaging, Cancer Screening, and High-Precision Radiotherapy, 1-17 Toyosuna, Mihama-ku, Chiba 261-0024, Japan; Radiation Safety and Quality Assurance Division, National Cancer Center Hospital, 5-1-1 Tsukiji, Chuo-ku, Tokyo 104-0045, Japan; Department of Radiation Oncology, Saitama Cancer Center, 780, Ooazakomuro, Ina, Saitama 362-0806, Japan; Department of Radiation Sciences, Komazawa University, 1-23-1 Komazawa, Setagaya, Tokyo 154-8525, Japan; Department of Radiation Oncology, St. Marianna University Hospital, 2-16-1, Sugao, Miyamae-ku, Kawasaki City, Kanagawa 216-8511, Japan; Department of Radiation Therapy, Ibaraki Prefectural Central Hospital, 6528 Koibuchi, Kasama City, Ibaraki 309-1793, Japan; Department of Radiation Therapy, Tokyo Medical University Hospital, 6-7-1 Nishishinjuku, Shinjuku-ku, Tokyo 160-0023, Japan; Department of Radiation Oncology, Aichi Cancer Center Hospital, 1-1, Kanokoden, Chikusa-ku, Aichi 464-8684, Japan; Department of Health Sciences, Faculty of Life Sciences, Kumamoto University, 4-24-1 Kuhonji, Chuo-ku, Kumamoto 862-0976, Japan; Department of Radiology, Hamamatsu University School of Medicine, 1-20-1 Handayama, Higashi-ku, Hamamatsu, Shizuoka 431-3192, Japan

**Keywords:** diagnostic reference levels, computed tomography dose index, dose–length product, radiotherapy, national survey, planning computed tomography

## Abstract

Previous studies have primarily focused on quality of imaging in radiotherapy planning computed tomography (RTCT), with few investigations on imaging doses. To our knowledge, this is the first study aimed to investigate the imaging dose in RTCT to determine baseline data for establishing national diagnostic reference levels (DRLs) in Japanese institutions. A survey questionnaire was sent to domestic RT institutions between 10 October and 16 December 2021. The questionnaire items were volume computed tomography dose index (CTDI_vol_), dose–length product (DLP), and acquisition parameters, including use of auto exposure image control (AEC) or image-improving reconstruction option (IIRO) for brain stereotactic irradiation (brain STI), head and neck (HN) intensity-modulated radiotherapy (IMRT), lung stereotactic body radiotherapy (lung SBRT), breast-conserving radiotherapy (breast RT), and prostate IMRT protocols. Details on the use of motion-management techniques for lung SBRT were collected. Consequently, we collected 328 responses. The 75th percentiles of CTDI_vol_ were 92, 33, 86, 23, and 32 mGy and those of DLP were 2805, 1301, 2416, 930, and 1158 mGy·cm for brain STI, HN IMRT, lung SBRT, breast RT, and prostate IMRT, respectively. CTDI_vol_ and DLP values in institutions that used AEC or IIRO were lower than those without use for almost all sites. The 75th percentiles of DLP in each treatment technique for lung SBRT were 2541, 2034, 2336, and 2730 mGy·cm for free breathing, breath holding, gating technique, and real-time tumor tracking technique, respectively. Our data will help in establishing DRLs for RTCT protocols, thus reducing imaging doses in Japan.

## INTRODUCTION

In radiotherapy (RT), image registration for position matching is essential for irradiating a target with treatment beam. Image-guided radiotherapy (IGRT) using an image guidance device is being routinely performed [1–4], and this significantly improves position accuracy during irradiation. The combined use of IGRT with technologies, such as stereotactic irradiation (STI), stereotactic body radiotherapy (SBRT), and intensity-modulated radiotherapy (IMRT), allows further concentration of the dose at the target and reduces the dose exposure to normal tissues.

Radiotherapy planning computed tomography (RTCT), which provides a reference image for the RT, is important for performing these highly accurate techniques. 4DCT provides the benefit of accurately estimating the position of a moving target during respiration [5]. However, 4DCT increases the radiation exposure dose for the entire RTCT scan protocol. Previous studies have focused on RTCT imaging quality [6], with few studies evaluating imaging doses in RTCT.

Excessive radiation exposure to non-target areas increases the risk of developing secondary cancer [7]. Therefore, we should reduce exposure doses as low as reasonably achievable to minimize the risk. The International Commission on Radiological Protection (ICRP) recommends the use of medical procedures and optimal radiological protection to manage patient radiation doses and avoid unnecessary radiation exposure in medical imaging [8–13]. The IEC60601-2-44 [14] requires the display of CT dose metrics, such as the volume computed therapy dose index (CTDI_vol_) and dose–length product (DLP) [8, 15], as proof of mechanical performance.

In recent years, diagnostic reference levels (DRLs) for imaging diagnoses have been reported. In 2012, Fukushima *et al.* [16] collected data focused on the DLP aimed at establishing Japan’s DRLs. In 2014, Matsunaga *et al.* published the 75th percentile of the CTDI_vol_ and effective doses for some CT examinations on adults and 5-year-old children, based on a nationally distributed questionnaire in Japan [17, 18]. Subsequently, Japan’s DRLs were published in 2015 [19] and updated in 2020 [20, 21]. There have been reports regarding DLRs in several countries overseas [22–27]. Those studies or guidelines reported representative doses (CTDI_vol_ and DLP for CT) for standard-sized patients obtained from many institutions in each country and region. For RT, the UK’s DRLs including RTCT were authorized by the Health Agency in UK [26]. However, such information has not yet been established for radiotherapeutic equipment in Japan. To our knowledge, no domestic research directly related to DRLs for RT exists; therefore, DRLs concept may be poorly understood in Japan. According to the guidelines on DRLs provided in ICRP135 [13], the concept of DRLs should also be applied to RT. Therefore, in this study, we examined the RTCT dose to obtain reference data for establishing DRLs in Japan. Additionally, the study investigated the variations in RTCT imaging dose by collecting data from numerous institutions regarding five popular treatment protocols.

## MATERIALS AND METHODS

### Questionnaire summary

The questionnaire, designed using a free online survey system (Google Forms), was distributed via the mailing list system of the Japanese Society for Radiation Oncology (JASTRO). The ethics review board of Tokyo Metropolitan Bokutoh Hospital (IRB02-097) approved this study. The present study was conducted in collaboration with JASTRO, the Diagnostic Radiation Subcommittee of the Japan Society of Medical Physics Measurement Committee and the Society’s Radiation Protection Committee between 10 October and 16 December 2021, with a focus on domestic RT institutions. The target for data collection was the RTCT at each institution. The main items in the questionnaire included acquisition parameters for the treatment protocol of brain STI, head and neck (HN) IMRT, lung SBRT, breast-conserving radiotherapy (breast RT) excluding after mastectomy, and prostate IMRT. As a result of a preliminary review by all co-authors in the present study, these protocols are commonly practiced at many institutions, so we expected many responses. Information on the use of innovative applications to reduce the imaging dose, including auto exposure control (AEC) and image-improving reconstruction options (IIRO), such as iterative approximation [28, 29] or artificial intelligence [29–31], was also obtained. Median values of the dose indices (CTDI_vol_ and DLP) were obtained for three to five cases at each site. A summary of the main questionnaire is presented in Supplementary Table 1.

### Calculating 50th and 75th percentiles of the volume computed tomography dose index and dose–length products in radiotherapy planning computed tomography

In ICRP 135 [13], an example is provided on data collection in the 50–90 kg range to obtain DRLs for adults, who were assumed to have an average body weight of 70 kg. However, the patient population undergoing cancer treatment differs from those in diagnostic fields. Therefore, in this questionnaire, estimation of the exposure dose was limited to adults, who were assumed to have an average 60 kg body weight (range, 40–80 kg) in the population. To identify the CTDI_vol_ and DLP, which are exposure dose indicators in CT, an average of five cases (at least three cases) from each site, excluding body weights outside the 40–80 kg range, was considered. The phantom sizes were also collected for each protocol. To calculate the unified CTDI_vol_ and DLP for a phantom size of 16 cm for head STI and 32 cm for the others, the values obtained from each institution were converted using the approximations described in a previous study [32]. Finally, the 50th and 75th percentiles of the CTDI_vol_ and DLP were calculated. In the subanalysis, the data were divided into groups with or without AEC and IIRO. The differences between the average CTDI_vol_ values were evaluated separately for each protocol. Furthermore, the 75th percentiles of DLP categorized by motion-management techniques for lung SBRT were compared, and similarly, the 75th percentiles of DLP categorized by different imaging ranges of 4DCT for lung SBRT were compared.

## RESULTS

### Questionnaire summary

The survey received 328 responses from various domestic RT institutions, and the response rate from a total of 759 RTCTs [33] in Japan was 43%. Table 1 presents a summary of the questionnaire responses, detailing the distribution of CT scanners. Among the respondents, the top three CT manufacturers were CANON (CANON Medical, Tokyo, Japan) with 157 scanners, GE (General Electric Systems, Milwaukee, WI, USA) with 89 scanners, and Siemens (Siemens, Munich, Germany) with 74 scanners (Table 1). The total number of responses for treatment protocol, including brain STI, HN IMRT, lung SBRT, breast RT, and prostate IMRT, was 197, 194, 227, 304, and 231, respectively (Table 2). Furthermore, the questionnaire revealed that the majority of the scanners (98.5%) were capable of displaying the CT exposure dose. The AEC was used in >61.3% of all the clinical protocols. Contrast-enhanced (CE) CT was additionally performed for brain STI and HN IMRT in 21.0 and 28.9% of the institutions, respectively. For lung SBRT, 56.3% of the institutions performed irradiation under free breathing, while 79.0% performed 4DCT more than or equal to once (Table 3). Fifty-seven percent of the institutions conducted pre-scans for prostate IMRT.

**Table 1:** Questionnaire summary of basic information in each computed tomography (CT) scanner

Property	Answer	*n* = 328 (%)
Manufacturer	CANON GE Siemens Philips Hitachi Other	157 (47.9%) 89 (27.1%) 74 (22.6%) 4 (1.2%) 3 (0.9%) 1 (0.3%)
Number of CT detector array	≤4 6–16 20–64 80–256 320	12 (3.7%) 171 (52.1%) 91 (27.7%) 45 (13.7%) 9 (2.7%)
Year installed	≤2011 2012–2013 2014–2015 2016–2017 2018–2019 2020–2021	77 (23.5%) 54 (16.5%) 62 (18.9%) 33 (10.1%) 52 (15.9%) 50 (15.2%)
Intent the use of CT	Planning only Both planning and diagnostic	231 (70.4%) 97 (29.6%)
Capability of CTDI_vol_ displayed	Yes	323 (98.5%)
Capability of DLP displayed	Yes	323 (98.5%)
Motivation to use iterative reconstruction based on AI	Reducing dose Improving image quality Both Not used Not installed	37 (11.3%) 21 (6.4%) 69 (21.0%) 68 (20.7%) 133 (40.6%)
Use of metal artifact reduction	Always use Use if a metal is implanted Not used Not installed	13 (4.0%) 139 (42.4%) 30 (9.2%) 146 (44.5%)
Use of dual-energy CT	Used Not used Not installed	8 (2.5%) 59 (18.1%) 259 (79.4%)

**Table 2:** Questionnaire summary of acquiring conditions and exposure doses in computed tomography scan for five clinical protocols

Property		Brain STI	HN IMRT	Lung SBRT	Breast RT	Prostate IMRT
Operated		197/328 (60.0%)	194/328 (59.1%)	227/328 (69.2%)	304/328 (92.7%)	231/328 (70.4%)
Mode of slice thickness		1.0 (mm)	2.0 (mm)	2.0 (mm)	2.0 (mm)	2.0 (mm)
Mode of tube voltage	First: Second:	120 kV (92.3%) 130 kV (3.1%)	120 kV (92.7%) 130 kV (3.6%)	120 kV (93.3%) 130 kV (3.6%)	120 kV (93.0%) 130 kV (4.0%)	120 kV (92.1%) 130 kV (3.1%)
Use of AEC		119/194 (61.3%)	149/193 (77.2%)	162/225 (72.0%)	226/302 (74.8%)	175/228 (76.1%)
Plain or CE imaging	Plain only: CE only: Both:	129/195 (66.2%) 25/195 (12.8%) 41/195 (21.0%)	116/194 (59.8%) 22/194 (11.3%) 56/194 (28.9%)	N/A	302/303 (99.7%) 1/303 (0.3%) 0/303 (0.0%)	226/230 (98.3%) 0/303 (0.0%) 4/230 (1.7%)
Mode of total number of CT series	First: Second:	1 series (81.1%) 2 series (14.6%)	1 series (67.7%) 2 series (27.1%)	2 series (29.6%) 3 series (24.5%)	1 series (91.7%) 2 series (3.7%)	1 series (49.3%) 2 series (37.8%)
CTDI_vol_ (mGy)^a^	50th percentile: 75th percentile:	68 92	20 33	50 86	16 23	21 32
DLP (mGy·cm)^a^	50th percentile: 75th percentile:	1851 2805	808 1301	1563 2416	629 930	802 1158
Phantom size used to calculate CTDI_vol_ and DLP		16 cm (60.5%) 32 cm (39.5%)^b^	16 cm (20.0%)^c^ 32 cm (80.0%)	32 cm (100.0%)	32 cm (100.0%)	32 cm (100.0%)

^a^See Figs 1 and 2.

^b^All data were converted to values equivalent to a phantom size of 16 cm when calculating CTDI_vol_ and DLP.

^c^All data were converted to values equivalent to a 32 cm phantom size when calculating CTDI_vol_ and DLP.

**Table 3:** Responses for the extra Section III for lung SBRT and prostate IMRT

Protocol	Property	Answer	*N* (%)
Lung SBRT	Technique of motion management	Free breath Breath holding Gating technique Dynamic tracking technique Other	126 (56.3%) 36 (16.1%) 31 (13.8%) 18 (8.0%) 13 (5.8%)
	Range of 4DCT	Whole Restricted in all lung Restricted around tumor Do not acquire or do not have	47 (21.0%) 31 (13.8%) 98 (43.8%) 48 (21.4%)
	Number of 4DCT series	0 1 2 Over 3 times	47 (21.0%) 147 (65.6%) 22 (9.8%) 6 (2.7%)
Prostate IMRT	Intent of pre-scan to check the condition of rectum and bladder	Always acquiring Sometimes acquiring No	66 (28.7%) 65 (28.3%) 99 (43.0%)

### Volume computed tomography dose index and dose–length products in radiotherapy planning computed tomography

**Fig. 1:**
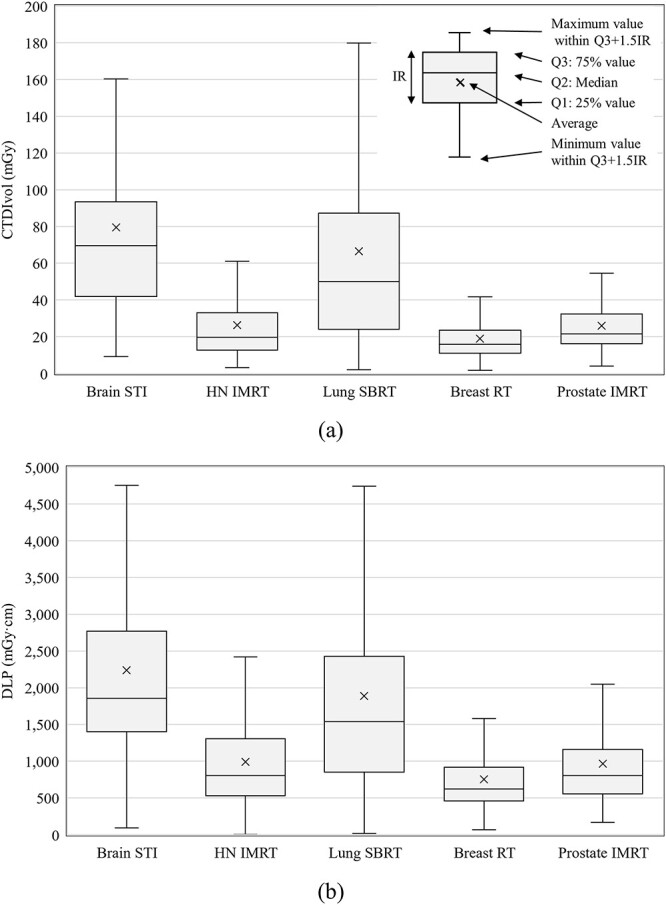
Overall results of (**a**) CTDI_vol_ and (**b**) DLP.

Details of the slice thickness, CTDI_vol_, and DLP statistics are presented in Table 2 and Fig. 1. The 75th percentiles of CTDI_vol_ were 92, 33, 86, 23, and 32 mGy and those of DLP were 2805, 1301, 2416, 930, and 1158 mGy·cm for brain STI, HN IMRT, lung SBRT, breast RT, and prostate IMRT, respectively. Furthermore, the slice thickness modes were 1.0, 2.0, 2.0, 2.0, and 2.0 mm for brain STI, HN IMRT, lung SBRT, breast RT, and prostate IMRT, respectively.

**Fig. 2:**
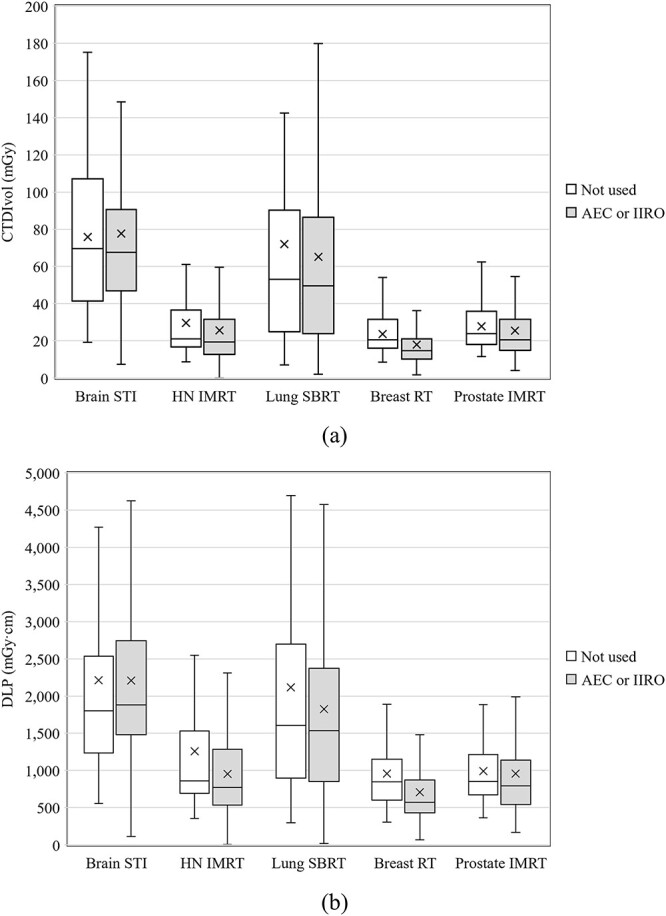
The (**a**) CTDI_vol_ and (**b**) DLP differences between groups with or without AEC and IIRO in brain STI, HN IMRT, lung SBRT, and prostate IMRT.

**Fig. 3:**
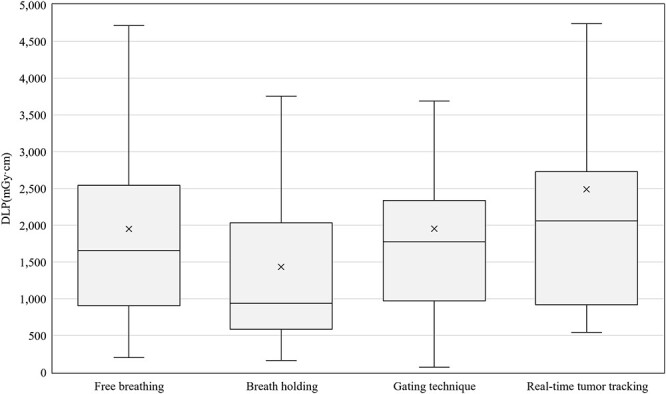
The DLP differences between motion management techniques for lung SBRT.

**Fig. 4:**
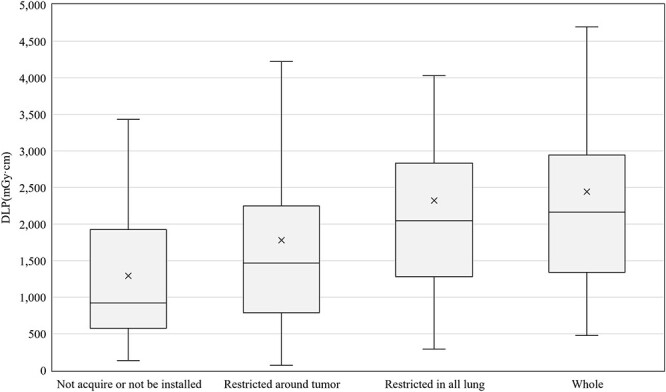
The DLP differences between different scan ranges of 4DCT for lung SBRT.

Figure 2 summarizes the average CTDI_vol_ differences between the groups with and without AEC and IIRO in each protocol. The 75th percentiles of CTDI_vol_ without AEC and IIRO were 107, 37, 90, 32, and 36 mGy and those of CTDI_vol_ with AEC or IIRO were 91, 32, 87, 21, and 32 mGy for brain STI, HN IMRT, lung SBRT, breast RT, and prostate IMRT, respectively. For breast RT, the value was reduced by 33.2% in the groups treated with AEC or IIRO. The 75th percentiles of the DLP in each treatment technique for lung SBRT were 2541, 2034, 2336, and 2730 mGy·cm for the free breathing, breath holding, gating, and real-time tumor tracking techniques, respectively (Fig. 3). The 75th percentiles of DLP in lung SBRT with 4DCT scan protocols were 1925, 2248, 2835, and 2946 mGy·cm for ‘not acquire or not be installed’, ‘restricted around tumor’, ‘restricted in all lung’ and ‘whole in planning range’, respectively (Fig. 4).

## DISCUSSION

To the best of our knowledge, this was the first study to estimate the exposure dose acquisitions for RTCT in Japan. Fortunately, 328 responses were obtained because the number of scanners that could be displayed for CTDI_vol_ and DLP was extremely high at 98%. We found that the general tendencies for the various scan protocols were that doses used for brain STI and lung SBRT were higher and had more variations than those used for other clinical protocols (Fig. 1). For brain STI, the following reasons were considered: (i) a phantom diameter of 16 cm was used to calculate the CTDI_vol_, (ii) thin slices (e.g. 1 mm) were needed, and (iii) tumor contrast in the brain was emphasized. Tumor contrast depends on the institution’s policy on whether to perform CE imaging and whether obtaining good tumor contrast was prioritized. Variation of the policies is assumed to be one of the reasons for the wide range in CTDI_vol_ and DLP. For the brain STI DLP, the reason for the group without AEC or IIRO having a lower 75th percentile than the group with AEC or IIRO was unclear (Fig. 2b). There were 42 responses in the former group and 133 in the latter; the former responses may have been slightly low in number. However, considering that the difference between the two was not large, it is also possible that AEC or IIRO were not effective for brain CT. For SBRT, the use of 4DCT or multiple scans to evaluate the range of tumor motion is a reason for increasing the dose and its variation. Additionally, there was a difference between the groups depending on the acquisition range of 4DCT and the technique used against respiratory movement in SBRT (Figs 3 and 4). Patient arm position is another factor in increasing dose variations. Bayer *et al.* [34] reported that the effective dose difference between arm-up and arm-down positioning was ~28%.

DRLs for RTCT were published in the United Kingdom (UK) in 2022 [24, 26]. Other countries, such as Ukraine [27] and Slovenia [25], have also reported domestic DRLs based on large amounts of data. In the UK report, the CTDI_vol_ and DLP of the brain and ‘head and neck’ were evaluated in a 16-cm phantom. The CTDI_vols_ of the brain, HN, breast, and prostate that were compared in this study were 50, 49, 10, and 16 mGy, respectively. The DLPs of the brain, HN, breast, and prostate were 1500, 2150, 390, and 570 mGy·cm, respectively. The CTDI_vol_ values in the UK DRLs in 2022 were 14 and 63 mGy for 3D and 4D lungs, respectively. The DLPs values were 550 and 1750 mGy·cm for 3D and 4D lungs, respectively. These values are comparable to or slightly lower than our data. However, our data cannot be simply compared with the UK DRLs for lung SBRT because our CTDI_vol_ and DLP data also included some static series in addition to the 4DCT series. Especially for the breast RT, the 75th percentile in our study was 2.38 times higher than that applied in the UK. We recognize that the RTCT imaging dose in breast RT is an indicator of future dose optimization processes because breast RT is basic, popular and easy to compare with other communities. Wood *et al.* [24] reported a close relationship between patient weight and CTDI_vol_, further emphasizing that body weights are important factor to consider and establish DRLs. Thus, simple comparisons would not be appropriate. However, we obtained 328 responses, which sufficiently exceeded the UK dataset number of 68. By referring to our data, institutions can objectively evaluate whether their data are appropriate. Furthermore, our findings will lead to a reduction in exposure doses in Japan.

In this study, we determined the population distribution of the exposure dose for RTCT and calculated it as part of establishing DRLs. However, the collected data were expressed as the median of the dose index based on three to five cases with a body weight of 40–80 kg at each institution. This differed from that of the DRL, wherein DRL was calculated for a standard body size. Furthermore, the CTDI_vol_ and DLP of RTCT cannot be compared with the DRL because the image quality, number and types of images and scan range or field of view required for RT are different. According to a review by Davis *et al.* [6], the quality of the images in RTCT must be within ±5 HU in the soft tissue. In addition, to calculate dose distribution using the treatment planning system, applying a CT value electron density conversion table to the RTCT image is necessary to obtain the density distribution information of the human body. Various imaging conditions may change HU, such as tube voltage, field of view, reconstruction algorithm, pitch, filter function and post-processing filter [35, 36]. Therefore, making significant changes to these imaging conditions is not recommended.

Generally, the CTDI_vol_ with either one or both the conditions of a head phantom (16 cm diameter) and a body phantom (32 cm diameter) is displayed on the CT scanner. However, the displayed content differs depending on the CT manufacturer and scan protocol. In RT, to unify the CT electron density conversion table applied to the dose calculation, the body scan protocol is also used for other small sites, such as the head or neck. Notably, this questionnaire captured the phantom diameter (16 or 32 cm) required to calculate CTDI_vol_. According to the American Association of Physicists Medicine (AAPM) Task Group 204 [32] and DRL2015 [19], the CTDI_vol_ in the 32 cm phantom is approximately half of that of the CTDI_vol_ in the 16-cm phantom. Recently, AAPM TG-204 introduced the concept of size-specific dose estimates (SSDEs), which has been proposed to evaluate CTDI_vol_ according to patient size [32, 37]. Alternatively, Saemi *et al.* [38] published a database with tools that estimated the exposure dose of each organ in RTCT using Monte Carlo simulations. This can be used to evaluate the risk of CT scans based on the dose delivered to each organ. Our study did not include SSDE or exposure dose data for each organ.

This study had some limitations. First, as the data collected in this study were the total exposure dose for the entire examination, they did not reflect the results of a single CT scan. Therefore, the more multiple scans are performed for one examination, the higher the total CTDI_vol_ and total DLP will be increased. This tendency will be more pronounced in lung SBRT, leading to greater variation between institutions. Second, information on the version of the CT dose calculation application was not collected. In future surveys, it will be necessary to investigate the CT versions to accurately understand the exposure dose in RTCT.

## CONCLUSION

In conclusion, an initial survey of the RTCT dose for brain STI, HN IMRT, lung SBRT, breast RT, and prostate IMRT was conducted to acquire reference data for establishing DRLs in Japan. The brain STI and, lung SBRT protocols resulted in increased CTDI_vol_ and DLP values owing to the thin slice thickness or acquisition of multiple series. Our data will be helpful in establishing DRLs for RT-planning CT protocols, which will lead to a reduction in imaging doses in Japan.

## Supplementary Material

Supplementary_Table_1_20230807_rrad098
