# Supplementary material for: Radiological imaging protection: a study on imaging dose used while planning computed tomography for external radiotherapy in Japan
Source: J Radiat Res. 2023 Dec 26;65(2):159–67. doi: 10.1093/jrr/rrad098 (PMC10959444; doi:10.1093/jrr/rrad098)
Supplement: Supplementary_Table_1_20230807_rrad098 [file supplementary_table_1_20230807_rrad098.docx]

**Supplementary Table 1. Questionnaire items**

| Questionnaire items |
| --- |
| 1.1 Facility name  1.2 Prefecture  1.3 Name of respondent |
| 2.1 CT manufacturer name  2.2 Number of CT slices used in treatment planning CT  2.3 Year of installation of treatment planning CT  2.4 Is the CT used for treatment planning exclusive to this purpose, or is it also used for diagnosis?  2.5 Is CTDI_vol_ displayed on the treatment planning CT?  2.6 Is DLP displayed on the treatment planning CT?  2.7 Is iterative reconstruction based on artificial intelligence technology used?  2.8 Is the metal artifact reduction reconstruction method used?  2.9 Is dual-energy CT used? |
| For brain stereotactic irradiation, head and neck IMRT, lung SBRT, breast conserving radiotherapy, and prostate IMRT,  3.1 Is the case performed?  3.2 Slice thickness (mm)  3.3 Tube voltage  3.4 Was AEC used?  3.5 Are simple and contrast-enhanced CT scans taken separately?  3.6 The total number of CT scans taken.  3.7 The median CTDI_vol_ (mGy) of the most recent five cases for all scan series.  3.8 The median DLP (mGy cm) of the most recent five cases for all scan series.  3.9 What is the phantom size used to calculate CTDI_vol_ and DLP?  3.10 If there are any questions that were difficult to answer, please write the reason, if possible.  Specialized questionnaire for lung SBRT,  3.11 The motion management technique frequently used at your facility.  3.12 How is the acquiring range for 4DCT set?  3.13 The total number of 4DCT  3.14 The total number of CT performed other than 4DCT (enter 0 if not performed).  Specialized questionnaire for prostate IMRT,  3. 15 Are pre-scans for confirmation of rectal and bladder capacity being performed?  3. 16 The total number of CT scans, including pre-scans. |
| 4.1 How did you feel about the length of the questionnaire?  4.2 If there are similar questionnaires in the future, would you be willing to participate?  4.3 If there was a field for the patient's weight in the questionnaire, would you be able to provide an answer?  4.4 What is an appropriate weight range according to you?  4.5 If there are any areas in this questionnaire that need improvement, please let us know.  4.6 Would you like to share any other comments. |

*Abbreviations*: CT, computed tomography; CTDI_vol_, computed tomography dose index volume; DLP, dose-length product; IMRT, intensity-modulated radiotherapy; SBRT, stereotactic body radiotherapy; AEC, auto-exposure control (AEC); CE, contrast-enhanced; 4DCT, four-dimensional CT.
